# Hospital Meetings

**Published:** 1906-04-21

**Authors:** 


### HOSPITAL MEETINGS.

### CITY OF LONDON HOSPITAL FOR DISEASES OF THE CHEST.

The chair was taken at the annual meeting of the governors of this hospital by Alderman Sir George Wyatt Truscott, Chairman of the Committee of Management.

The adoption of the annual report was moved by the Chairman, who remarked that the smallness of the attendance at the meeting testified to the contentment of the governors with the way in which the hospital was carried on. The report was a satisfactory one, in so far as it showed that during the period of great depression which had affected the City and the country generally the income of the hospital had been well maintained. But it could not be entirely satisfactory so long as they had closed beds in the hospital. They had accommodation for 164 patients, but in spite of all their efforts they were still obliged to close 24 beds for lack of funds. The Committee were very anxious for more money to enable them to do more work. The very important source of income, legacies, had fallen off considerably. There was no hospital that did more good than this one amongst the teeming population of East London. With regard to the administration of the hospital, they could rest assured that it was being well and economically carried on since it had the impress of the hospital's hall-marks, that is the approval of the committees of the three great funds, King Edward's Hospital Fund, Hospital Sunday Fund, and Hospital Saturday Fund. The Chairman then paid a tribute to the unremitting labours of Mr. George Masterman, Chairman of the House Committee. With regard to the open-air treatment, the very full account given in the report showed that in a large percentage of cases the treatment was most satisfactory, even in the fogs and damp of London. It was with deep regret that he mentioned the death of the late Mr. H. Edmund Gurney, who was the last of those gentlemen who had attended the first meeting of the institution in 1848. The Ladies' Association had done excellent work in the past year, and were now collecting funds towards the convalescent home which the Committee hoped would be the next scheme they could take in hand. During the past year the Committee had been compelled to sell out investments amounting to £3,000, partly to pay for the Nurses' Home and partly for other objects. They were very anxious to replace these funds at as early a date as possible.

